# People’s Health in People’s Hands: Dr. Abhay Bang and Dr. Rani Bang’s Pioneering Approach to Rural Healthcare in India

**DOI:** 10.7759/cureus.68345

**Published:** 2024-08-31

**Authors:** Umesh Kawalkar, Amar Mankar, Ravindra Gophane, Manoj S Patil

**Affiliations:** 1 Community Medicine, Government Medical College, Akola, IND; 2 Community Medicine, Datta Meghe Institute of Higher Education and Research, Wardha, IND; 3 Research and Development, Jawaharlal Nehru Medical College, Datta Meghe Institute of Higher Education and Research, Wardha, IND

**Keywords:** new born care, malnutrition, padma shri, maharashtra bhushan, historical vignette, arogyadoots

## Abstract

Dr. Abhay Bang and Dr. Rani Bang, a husband-and-wife team, have dedicated their lives to reforming healthcare in rural India. Their pioneering work in Gadchiroli, Maharashtra, through the Society for Education, Action, and Research in Community Health (SEARCH), has led to revolutionary interventions such as the Home-Based Newborn Care (HBNC) model, significantly reducing infant mortality rates. Their emphasis on women's health and community empowerment has revolutionized conventional healthcare practices and influenced global health policies. This review article sheds light on the historical context and lasting impact of their contributions to community-based healthcare and public health research.

## Introduction and background

Beginnings: the seeds of compassion

**Figure 1:**
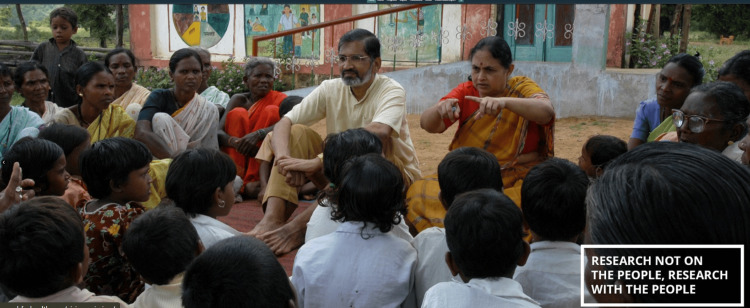
Dr. Abhay and Dr. Rani Bang interacting with the community Image Courtesy: Permission taken from Dr. Abhay & Dr. Rani Bang

Dr. Abhay Bang was born in Wardha, Maharashtra, in 1950. Gandhian ideology and philosophies were deeply rooted in his family traditions. His father, Thakurdas Bang, was not only a close associate of Mahatma Gandhi but also a prominent freedom fighter. From childhood, Abhay was surrounded by Gandhian ideology, which shaped his principles of selflessness and service to humanity. This environment sowed the seeds of compassion and a strong sense of social justice in Abhay from a young age [1]. Dr. Rani Bang was born in 1951 in Chandrapur with a different family background. Her father was a teacher, and her mother was a homemaker, instilling in her the values of hard work, education, and empathy. Rani excelled academically and developed a strong passion for medicine. Her path intersected with Abhay's at Nagpur University, where they were both pursuing medical studies. They shared a common vision of using their medical knowledge to serve the underprivileged. Their partnership, both personal and professional, was built on this shared commitment to social justice and healthcare [1]. Both completed their medical degrees from Government Medical College, Nagpur, Maharashtra. Dr. Abhay ranked first at Nagpur University in medical studies and Dr. Rani earned a gold medal in gynecology. After completing their medical education, Dr. Bang and his wife Rani (Figure 1) started their medical practice in the village of Kanhapur near Wardha. However, their initial approach of focusing solely on medical treatment proved to be insufficient. They recognized that effectively addressing the root causes of health issues required a comprehensive understanding of the social and economic contexts within the villages [1].

Abhay and Rani decided to pursue further studies in public health, enrolling at the Johns Hopkins University School of Public Health in the United States. Their time at Johns Hopkins was transformative, exposing them to global health challenges and innovative solutions. They were particularly inspired by community-based healthcare models in developing countries, which emphasized local involvement and sustainable practices.

At Johns Hopkins, they were deeply influenced by the work of public health pioneers like Dr. Carl Taylor, who advocated for primary healthcare as a means to achieve health equity. This exposure solidified their belief that meaningful healthcare interventions must be rooted in the communities they serve.

**Figure 2:**
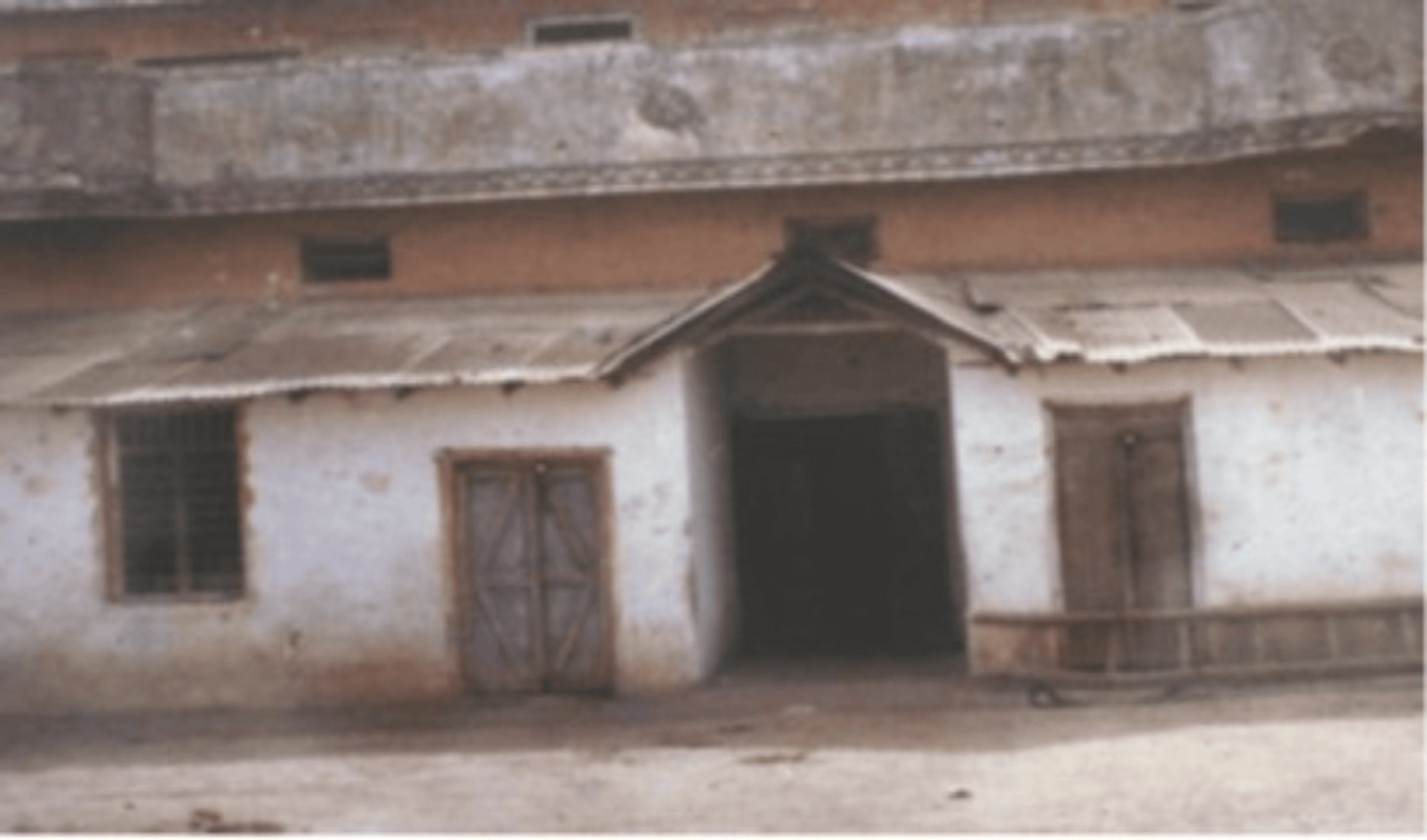
SEARCH office, 1986 SEARCH, Society for Education, Action, and Research in Community Health Image Courtesy: Permission taken from Dr. Abhay Bang

Abhay and Rani decided to pursue further studies in public health, enrolling at the Johns Hopkins University School of Public Health in the United States. Their time at Johns Hopkins was transformative, exposing them to global health challenges and innovative solutions. They were particularly inspired by community-based healthcare models in developing countries, which emphasized local involvement and sustainable practices. At Johns Hopkins, they were deeply influenced by the work of public health pioneers like Dr. Carl Taylor, who advocated for primary healthcare as a means to achieve health equity. This exposure solidified their belief that meaningful healthcare interventions must be rooted in the communities they serve [2]. With their new knowledge about public health and a strong desire to make a difference, Abhay and Rani came back to India in the early 1980s. They selected Gadchiroli, a faraway and poor tribal area in Maharashtra, to start their work. They created the Society for Education, Action, and Research in Community Health (SEARCH), Gadchiroli (Figure 2), and began their mission to learn about and help the tribal people with their health problems [1,2].

## Review

The Gadchiroli model: innovations in community healthcare

**Figure 3:**
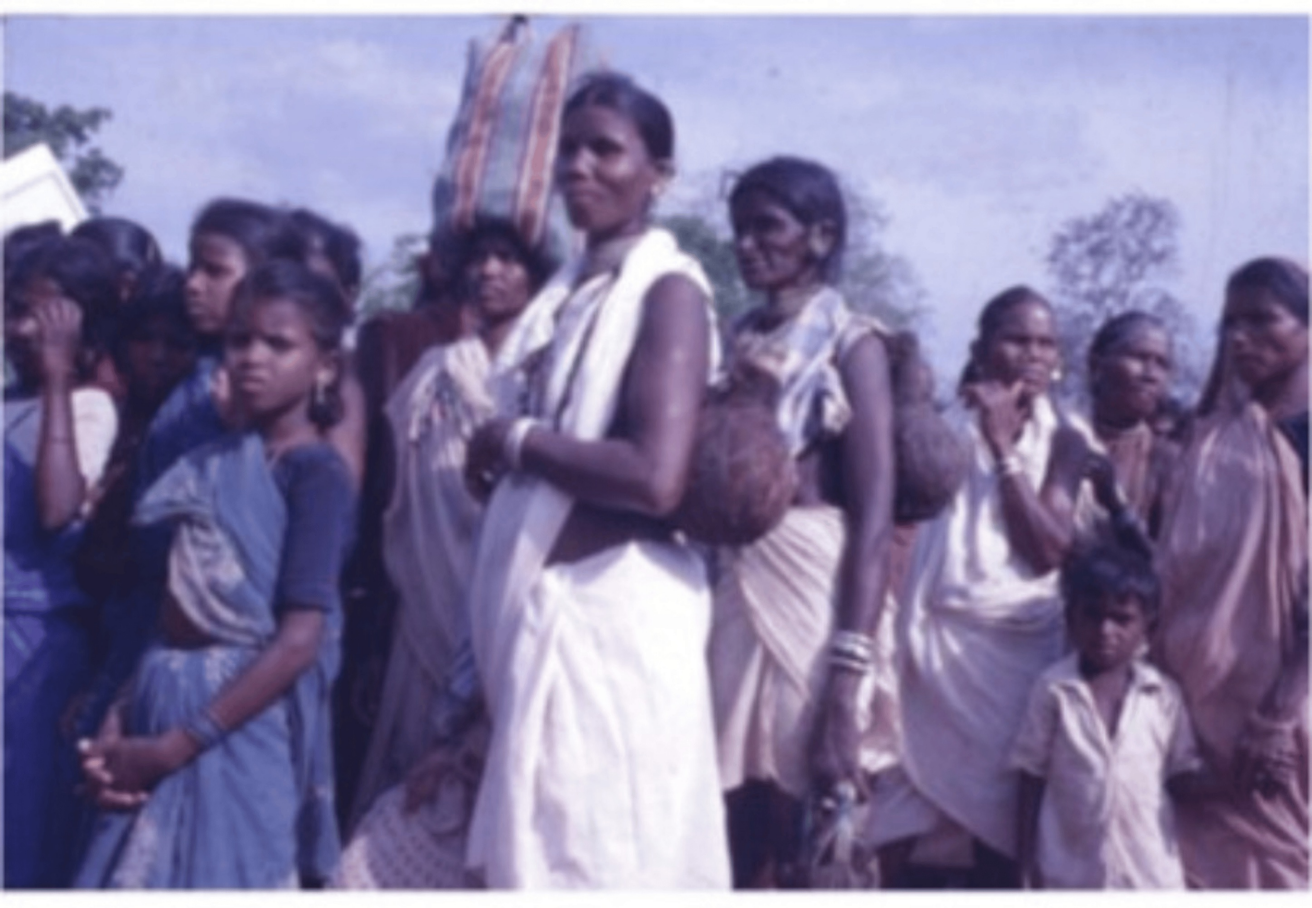
Tribal population in Gadchiroli (1986) Image Courtesy: Permission taken from Dr. Abhay & Dr. Rani Bang

The majority of the population in Gadchiroli lived below the poverty line, struggling to access basic necessities like food and shelter (Figure 3). The healthcare infrastructure was severely deficient, with hospitals few and far between, and often staffed by unqualified doctors. This lack of access was compounded by cultural barriers and fear, particularly among the tribal communities, who were hesitant to visit hospitals due to their unfamiliarity with the environment and the association of white uniforms with death in the community's perception [3]. The impact of these challenges was devastating, especially for the most vulnerable members of the community. The infant mortality rate was alarmingly high, and the state of maternal health was bad; the majority of women in the community were suffering from severe gynecological conditions but had no access to adequate treatment facilities [4]. Cultural and traditional practices, like keeping women who are menstruating away from others, and relying on traditional healers instead of doctors, made it even harder for people to get modern healthcare. These practices were deeply ingrained in the community and were passed down from generation to generation. During the monsoon seasons, the situation deteriorated due to a lack of infrastructure and incomplete bridges, leading to a loss of access to healthcare facilities [5]. This made it very hard for people to get the help they needed. Healthcare system that was inadequately serving the needs of the population [3].

The Gadchiroli experiment was a remarkable endeavor that transformed newborn healthcare in a challenging, resource-limited setting. The prevailing belief at the time was that only well-equipped hospitals could effectively treat sick newborns. However, Dr. Bang recognized the impracticality of this approach in rural Gadchiroli and became the driving force behind experimenting with alternative solutions [6]. Most births in the area occurred at home, often under less-than-ideal conditions. Expecting families to travel long distances with their sick newborns was simply unrealistic. The Bangs' solution was both innovative and empowering. They decided to train local women, known as Arogyadoots or "health messengers," to become skilled healthcare providers. Women, often with limited formal education, were trained to identify and manage common newborn health issues such as infections and hypothermia. They were given a simple kit containing essential tools like a bag-and-mask for helping babies breathe, a thermometer, and antibiotics [6,7]. The training program was designed to be practical and engaging, using games and role-playing to make complex medical knowledge easy to understand. The Bangs had complete faith in these women's abilities, and their trust was well-placed. The Arogyadoots proved to be quick learners, even impressing a panel of expert pediatricians with their skills [7,8].

**Figure 4:**
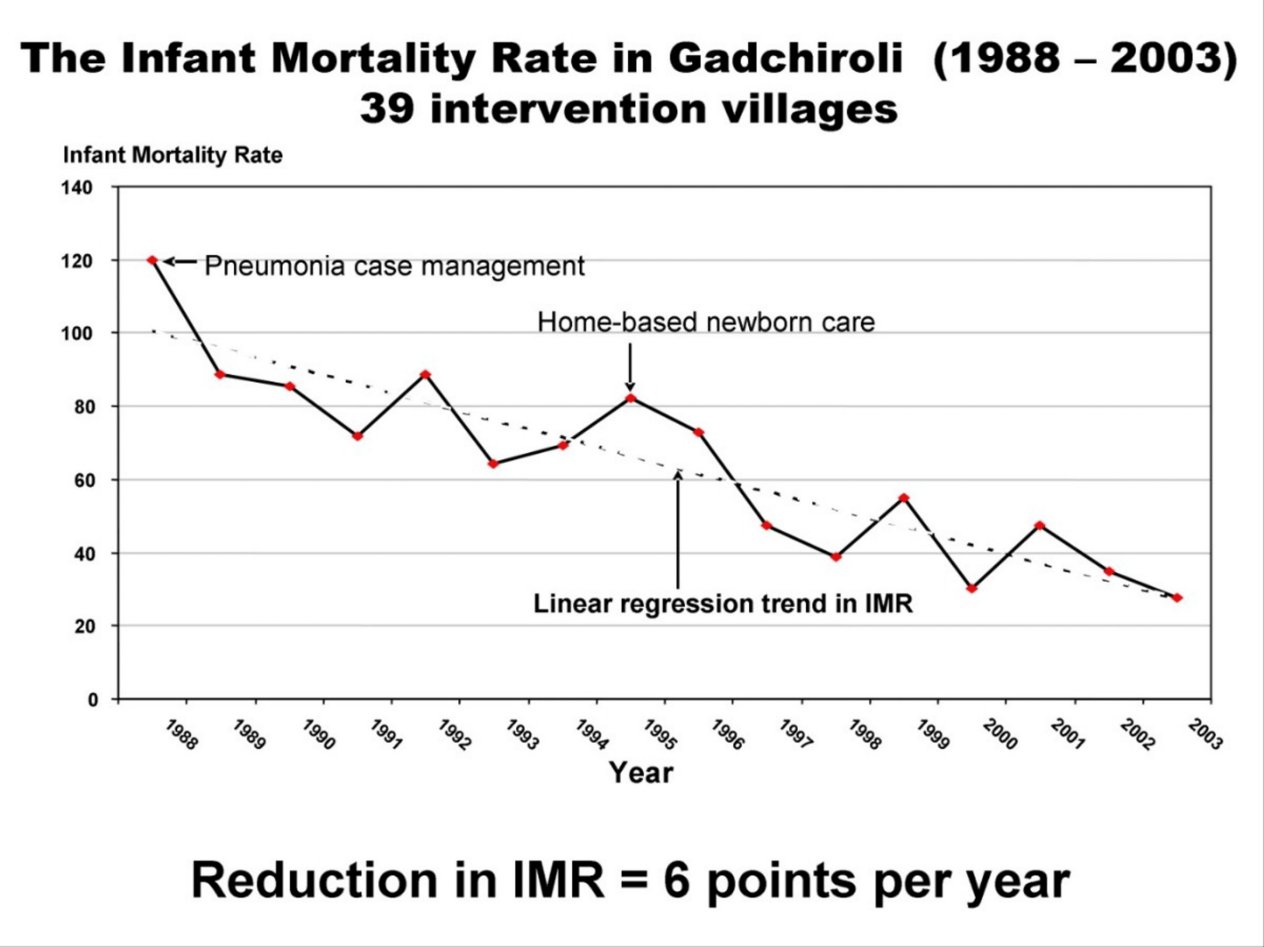
Trend of infant mortality rate after the Gadchiroli experiment (1988-2003) Image Courtesy: Permission taken from Dr. Abhay & Dr. Rani Bang; image adapted from https://searchforhealth.ngo/shaping-policy/

The impact of the Gadchiroli experiment was nothing short of extraordinary. The Arogyadoots, armed with their newfound knowledge and simple tools, had effectively turned countless homes into mini-intensive care units, saving countless lives [3]. The infant mortality rate, which was initially very high, significantly decreased. The Arogyadoots, the local women trained as healthcare providers, were able to successfully treat newborn infections, reducing the death rate from 17% to a mere 2.8%. They also administered thousands of injections without any complications, proving their competence. The overall newborn mortality rate in the areas where the program was implemented dropped by an impressive 62%. This achievement was particularly noteworthy considering that even a 10% reduction is considered significant in developed countries (Figure 4) [9]. The experiment demonstrated that it was possible to provide effective newborn care at a low cost and within the community. The Gadchiroli experiment was remarkably cost-effective. The cost of providing home-based neonatal care was just INR 350 per newborn or INR 8,000 per village. The cost to save one life was calculated at INR 7,000. If that saved child lived for 60 years, the cost per year of life saved would be a mere INR 115. Even more striking, if we consider only the economically productive years (estimated at 23 by the WHO), the cost to save one productive year of life was only INR 350. This was significantly lower than the cost estimates of other mother and child health programs, which ranged from INR 5,000 to INR 10,000 per productive year of life saved [8,9].

The Gadchiroli model proved that high-quality healthcare could be delivered at a fraction of the usual cost. The impact of the Gadchiroli experiment extended far beyond the borders of the district. The research findings were published in the prestigious medical journal, The Lancet, and garnered global attention. The study challenged the prevailing notion that specialized hospitals were essential for newborn care and highlighted the potential of community-based interventions. WHO, which had previously advised against treating newborns at home, began to take notice of these developments. The Indian Council for Medical Research (ICMR) planned to expand the Gadchiroli model into five states, recognizing its potential benefits. The funds were provided by the Bill and Melinda Gates Foundation to replicate the model in other developing countries. The Gadchiroli experiment's impact spread to countries in Asia and Africa, demonstrating the global applicability of this innovative approach. The experiment's success underscored the power of simple, community-based solutions in addressing critical healthcare challenges, proving that impactful change does not always require expensive technology or complex infrastructure [9-14].

Women health

Dr. Rani Bang's work in women's health was truly revolutionary. In Gadchiroli, she encountered a stark reality that shaped her approach. The majority of women in Gadchiroli suffered from gynecological problems, yet these issues were largely unaddressed by the existing healthcare system. The prevailing belief was that women in developing countries primarily required maternal health and family planning services. Dr. Bang's research on 650 women observed that more than half had gynecological problems. Surprisingly, almost all of them (92%) had at least one gynecological or sexual disease, with each woman having an average of 3.6 different conditions. Half of these problems were infections such as Trichomonas and Candida or bacterial vaginitis. Sadly, only a small number (8%) had ever seen a doctor for these issues. Driven by her findings, Dr. Bang did not just publish her research in prestigious journals like The Lancet, she actively implemented changes to address these issues [15,16]. She spearheaded community-based interventions, training local midwives and creating culturally sensitive programs that addressed the specific needs of these women. Through educational initiatives like the Women's Awareness and Health Fair, she broke down taboos surrounding women's health, fostering open dialogue and empowering women to seek care. Dr. Bang's work transcended mere treatment, it was about empowering women, giving them a voice and agency over their own health and bodies. Her efforts led to a paradigm shift in how women's health was perceived and addressed, impacting not only Gadchiroli but also influencing global health policies. She proved that even in resource-limited settings, significant improvements in women's health were possible through community-based, culturally relevant interventions [16,17].

Tribal health

Dr. Abhay and Dr. Rani Bang's work in Gadchiroli extended beyond maternal and child health to encompass the broader spectrum of tribal health concerns. They recognized that healthcare needed to be culturally sensitive and community-driven to be effective. One innovative approach was the annual tribal Health Mela or People's Health Assembly, a vibrant gathering that blended traditional festivities with health education. This provided a platform for open discussions about health challenges and fostered community ownership of solutions [2]. Malaria, a persistent threat in the region, was tackled through a community-based program. Local health workers and even traditional healers were trained in diagnosis and treatment, and the use of insecticide-treated bed nets was promoted. This multifaceted approach led to a significant reduction in malaria cases. The Bangs also confronted the pervasive issue of alcoholism, recognizing its detrimental impact on health and social well-being. They actively supported women-led movements against alcohol abuse, culminating in the successful implementation of prohibition in Gadchiroli. This underscored their understanding that health is inextricably linked to social and cultural factors. In addition, the Bangs tackled the root causes of ill health, such as poverty and malnutrition. The Bangs understood that healthcare alone is not enough to improve the lives of people living in poverty. They also supported sustainable ways for people to make a living and get enough food. Their approach to healthcare focused on respecting the local culture, empowering the community, and addressing the social and economic factors that affect health. This has made a real difference in the lives of the tribal people in Gadchiroli. Dr. Abhay Bang believes that research is very important, especially when it is done by living with and understanding the people you are trying to help. He thinks that this kind of research can lead to big changes and improvements in society [16,18].

Recognition

Dr. Abhay Bang and Dr. Rani Bang have been recognized with numerous prestigious awards and honors for their groundbreaking work in community health. Notably, both received the Padma Shri, one of India's highest civilian awards, in recognition of their distinguished service in the field of medicine and healthcare. They were also jointly honored with the Maharashtra Bhushan, the highest civilian award of the state of Maharashtra. TIME magazine acknowledged their global impact by naming them Global Health Heroes in 2005. Their work has also been lauded by various national and international organizations, including the WHO and UNICEF. These awards and recognitions underscore the profound and lasting impact of their contributions to community health and public health research [2,19].

## Conclusions

The Gadchiroli experiment, led by Dr. Abhay and Dr. Rani Bang, has shown us that it is possible to bring about big changes in healthcare, even in places with limited resources. They proved that by training local women and working closely with the community, we can save the lives of many newborns. Their work also highlighted the importance of women's health and showed us that simple, affordable solutions can make a huge difference. The Bangs's dedication and innovative spirit continue to inspire people around the world, reminding us that everyone deserves access to quality healthcare, no matter where they live.
